# Venetoclax plus low-dose cytarabine in patients with newly diagnosed acute myeloid leukemia ineligible for intensive chemotherapy: an expanded access study in Japan

**DOI:** 10.1093/jjco/hyad027

**Published:** 2023-04-04

**Authors:** Noboru Asada, Jun Ando, Satoru Takada, Chikashi Yoshida, Kensuke Usuki, Atsushi Shinagawa, Kenichi Ishizawa, Toshihiro Miyamoto, Hiroatsu Iida, Nobuaki Dobashi, Sumiko Okubo, Hideyuki Honda, Tomomi Soshin, Yasuko Nishimura, Atsuko Tsutsui, Harumi Mukai, Kazuhito Yamamoto

**Affiliations:** Department of Hematology and Oncology, Okayama University Hospital, Okayama, Japan; Department of Hematology, School of Medicine, Juntendo University, Tokyo, Japan; Leukemia Research Center, Gunma Saiseikai Maebashi Hospital, Maebashi, Japan; Department of Hematology, National Hospital Organization Mito Medical Center, Ibaraki-machi, Japan; Department of Hematology, NTT Medical Center Tokyo, Tokyo, Japan; Department of Internal Medicine, Hitachi General Hospital, Hitachi, Japan; Department of Internal Medicine III, Yamagata University Faculty of Medicine, Yamagata, Japan; Department of Medicine and Biosystemic Science, Kyushu University Graduate School of Medical Sciences, Fukuoka, Japan; Department of Hematology, National Hospital Organization Nagoya Medical Center, Nagoya, Japan; Division of Clinical Oncology/Hematology, The Jikei University Daisan Hospital, Tokyo, Japan; Department of Hematology and Cell Therapy, AbbVie GK, Osaka, Japan; Department of Hematology and Cell Therapy, AbbVie GK, Tokyo, Japan; Department of Hematology and Cell Therapy, AbbVie GK, Tokyo, Japan; Department of Hematology and Cell Therapy, AbbVie GK, Tokyo, Japan; Department of Hematology and Cell Therapy, AbbVie GK, Tokyo, Japan; Department of Hematology and Cell Therapy, Abbvie Inc., Singapore; Department of Hematology and Cell Therapy, Aichi Cancer Center, Nagoya, Japan

**Keywords:** acute myeloid leukemia, venetoclax, low-dose cytarabine, expanded access study, tumor lysis syndrome

## Abstract

**Background:**

In a Phase 3 international clinical trial (VIALE-C), venetoclax plus low-dose cytarabine improved the response rate and overall survival versus placebo plus low-dose cytarabine in patients with newly diagnosed acute myeloid leukemia who were ineligible for intensive chemotherapy. After the enrollment period of VIALE-C ended, we conducted an expanded access study to provide preapproval access to venetoclax in combination with low-dose cytarabine in Japan.

**Methods:**

Previously, untreated patients with acute myeloid leukemia who were ineligible for intensive chemotherapy were enrolled according to the VIALE-C criteria. Patients received venetoclax (600 mg, Days 1–28, 4-day ramp-up in Cycle 1) in 28-day cycles and low-dose cytarabine (20 mg/m^2^, Days 1–10). All patients took tumor lysis syndrome prophylactic agents and hydration. Safety endpoints were assessed.

**Results:**

Fourteen patients were enrolled in this study. The median age was 77.5 years (range = 61–84), with 78.6% over 75 years old. The most common grade ≥ 3 treatment-emergent adverse event was neutropenia (57.1%). Febrile neutropenia was the most frequent serious adverse event (21.4%). One patient developed treatment-related acute kidney injury, leading to discontinuation of treatment. Two patients died because of cardiac failure and disease progression that were judged not related to study treatment. No patients developed tumor lysis syndrome.

**Conclusions:**

The safety outcomes were similar to those in VIALE-C without new safety signals and were well managed with standard medical care. In clinical practice, more patients with severe background disease are expected, in comparison with in VIALE-C, suggesting that it is important to carefully manage and prevent adverse events.

## Teaser

This expanded access study showed a manageable safety profile of venetoclax plus low-dose cytarabine in Japanese patients with untreated acute myeloid leukemia who were ineligible for standard intensive induction chemotherapy.

## Introduction

The standard treatment strategy for newly diagnosed acute myeloid leukemia (AML) is an intensive curative chemotherapy, and a combination of cytarabine (AraC) and anthracycline is recommended as remission induction therapy. However, many AML patients are ineligible for intensive therapy because of advanced age or co-morbidities (1–3). Treatment options are limited for these patients, especially older patients, who account for a large proportion of patients with newly diagnosed AML (1,4,5). According to the guidelines of the Japanese Society of Hematology at the time of study initiation, the only recommended treatment for older patients with AML, in whom standard therapy is unsuitable but who are treatable, is low-dose AraC (LDAC) or participation in a clinical study (6).

Venetoclax is an orally available, small-molecule selective B-cell leukemia/lymphoma-2 inhibitor (7,8). In two placebo-controlled Phase 3 trials, the safety and efficacy of venetoclax-based therapy were confirmed in treatment-naive patients with AML who were ineligible for intensive chemotherapy owing to advanced age or co-morbidities. In the VIALE-A study, the venetoclax plus azacitidine (AZA) arm demonstrated significantly better outcomes compared with the placebo plus AZA arm (9). In the VIALE-C study, the venetoclax plus LDAC arm did not meet its primary endpoint of a statistically significant improvement in overall survival compared with the placebo plus LDAC arm (10). In the 6-month follow-up analysis of the VIALE-C study, the addition of venetoclax to LDAC increased the rates of complete remission (CR) and CR with incomplete blood count recovery (CRi) compared with the control arm (48 vs. 13%; *P* < 0.001) and extended median overall survival [8.4 vs. 4.1 months (hazard ratio = 0.70; *P* = 0.04)] (10,11). In the subgroup analysis, venetoclax plus LDAC was well tolerated in Japanese patients (5).

In November 2018, the US Food and Drug Administration granted accelerated approval for venetoclax in combination with AZA, decitabine or LDAC for the treatment of newly diagnosed AML in adults aged ≥75 years or who have co-morbidities that preclude the use of intensive induction chemotherapy. The expanded access study (EAS) framework (Japanese compassionate use program) was established in January 2016. This framework can provide preapproval access to unapproved or off-label drugs for patients under the following conditions: the target disease is serious and life-threatening with no effective therapy available; the drug, either unapproved or off-label, is under development in Japan and is in the final stage of development, that is, the pivotal trial (confirmatory trial for new drug application) has ended or patient enrollment in the trial has finished (12). A supplemental new drug application for venetoclax in AML was submitted in June 2020. Owing to the limited treatment options available for patients with AML who are not candidates for intensive chemotherapy, this EAS was conducted to support the use of venetoclax until approval. The VIALE-C regimen was adopted to provide venetoclax treatment to a broader range of patients, including those who had been pretreated with hypomethylating agents, such as AZA, which is in line with the purpose of the EAS as opposed to the VIALE-A study where those patients were excluded. In March 2021, venetoclax was approved for the treatment of AML by the Ministry of Health, Labour and Welfare of Japan based on results of the VIALE-A and VIALE-C studies. Here, we aim to present the safety results of venetoclax plus LDAC in Japanese patients who were ineligible for intensive chemotherapy in the EAS.

## Patients and methods

### Study design

This study was a single-arm, open-label, multicenter, EAS of venetoclax in combination with LDAC in newly diagnosed patients with AML who were ineligible for intensive induction therapy in Japan. The primary objective was to provide a treatment option with venetoclax plus LDAC to eligible patients in the EAS prior to the approval of venetoclax by the Ministry of Health, Labour and Welfare in Japan. There were no efficacy endpoints and only safety was assessed, but bone marrow and disease assessment were conducted at the investigator’s discretion to evaluate the disease condition based on patients’ physical findings, peripheral blood counts and/or bone marrow examination during study treatment. The protocol and informed consent form were reviewed and approved by an independent ethics committee/institutional review board at each site before initiation. All patients provided written informed consent before participating. The study was conducted in accordance with the International Council for Harmonization requirements, Good Clinical Practice guidelines and the Declaration of Helsinki.

### Patients

This study enrolled the following patients and had the identical eligibility criteria as the VIALE-C study. Eligible patients were adults (≥18 years old) with newly diagnosed AML according to World Health Organization criteria (13). Patients were considered to be ineligible for standard induction therapy if they were aged ≥75 years or with the presence of at least one of the following: Eastern Cooperative Oncology Group performance status (PS) 2 or 3; cardiac history of congestive heart failure requiring treatment or ejection fraction ≤50% or chronic stable angina; diffusion capacity of the lung for carbon monoxide ≤65% or forced expiratory volume in 1 second ≤65%; creatinine clearance of ≥30 ml/min to <45 ml/min; total bilirubin >1.5 to ≤3.0 times the upper limit of normal or other co-morbidities deemed incompatible with standard intensive chemotherapy.

### Treatment

**Figure 1:**
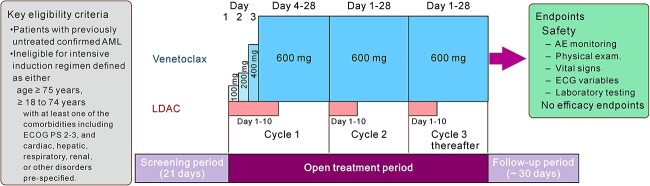
Design of the expanded access study. AE, adverse event; ECG, electrocardiogram; LDAC, low-dose cytarabine; AML, acute myeloid leukemia; ECOG, Eastern Cooperative Oncology Group; PS, performance status.

All patients received venetoclax 600 mg orally once a day or daily (QD) on Days 1–28 in combination with LDAC 20 mg/m^2^ subcutaneously on Days 1–10 in each 28-day treatment cycle, except for the first cycle (Cycle 1). In Cycle 1, venetoclax dosing began at 100 mg on Day 1 of the cycle and was then increased stepwise over 4 days (ramp-up period) to reach the target dose of 600 mg (100, 200, 400 and 600 mg). Treatment with the study drugs was continued until progressive disease (PD), unacceptable toxicity or other pre-established treatment discontinuation criteria were met, or until venetoclax was commercially available after its approval. All patients were followed up for 30 days after the last dose of venetoclax (Fig. 1).

Tumor lysis syndrome (TLS) prophylaxis and monitoring were implemented for all patients during the study as TLS risk mitigation measures. Specifically, all patients (i) were hospitalized prior to the initial dose of study treatment for at least 24 hours after reaching the target dose of venetoclax in Cycle 1 to monitor for TLS; (ii) received a uric acid reducing agent and hydration prior to and during the ramp-up period and (iii) underwent blood sampling for TLS chemistry tests, including calcium, inorganic phosphorus, potassium, uric acid and creatinine on Day 1 of each cycle and each day during the ramp-up period within 4 hours prior to dosing and 6–8 hours post-dosing of the study drug. Anti-infective prophylaxis for viral, fungal, bacterial or pneumocystis infections was required for patients with an absolute neutrophil count of <500/μl.

### Assessments

Safety evaluations were performed in enrolled patients throughout the study, including adverse event (AE) monitoring, physical examination, vital sign measurement, variables in electrocardiogram/two-dimensional echocardiogram/multi-gated acquisition scans and clinical laboratory testing (hematology, chemistry, liver functions and urinalysis) as measures of safety and tolerability for the entire study duration.

Treatment-emergent AEs (TEAEs) were defined as those that occurred between the first dose of the study drug until 30 days after the last dose of the study drug. AEs were graded according to the National Cancer Institute Common Terminology Criteria for Adverse Events Version 4.03.

Disease assessments were conducted at the discretion of the investigator according to patients’ physical findings, peripheral blood counts and/or bone marrow assessment during the study treatment, mainly at screening, the end of Cycle 1 and every three cycles thereafter. Clinical responses were defined according to the modified International Working Group Criteria for AML (14), and PD was defined as per European Leukemia Net recommendations (15).

Transfusion independence was defined as a period of at least 56 consecutive days with no red blood cell or platelet transfusion during the evaluation period. The post-baseline transfusion evaluation period was from the first dose of the study drug to the last dose of the study drug plus 30 days, PD, confirmed morphological relapse or death, whichever occurred earlier.

### Statistical methods

The sample size was not determined statistically. Safety was assessed through reported TEAEs, serious AEs (SAEs), AEs leading to discontinuation, death or changes in laboratory and vital sign parameters.

## Results

### Patients

Patient demographics and clinical characteristics are summarized in Table 1. This study was conducted at 11 sites in Japan between 5 October 2020 and 13 May 2021. Eighteen patients were screened, among whom 14 patients with AML were enrolled and received venetoclax in combination with LDAC; 4 patients were excluded for reasons of not meeting the eligibility criteria (*n* = 3) or early death before enrollment (*n* = 1). The median age was 77.5 years, 11 patients (78.6%) were ≥ 75 years old, 1 patient was 61 years old with moderate hepatic impairment (total bilirubin >1.5 to ≤3.0 upper limit of normal) and the other 2 patients were 70 and 72 years old with co-morbidities that the physicians judged to be incompatible with intensive chemotherapy. Most patients had Eastern Cooperative Oncology Group PS 0–1: seven patients (50.0%) were PS 0 and six patients (42.9%) were PS 1. Secondary AML was reported in 8 of 14 patients (57.1%), among whom 7 patients (87.5%) had myelodysplastic syndrome (MDS) overt AML. Five patients (35.7%) had a treatment history of AZA. Blast counts in bone marrow (<30%) were reported for 8 of 13 patients (61.5%), who were diagnosed with AML according to the World Health Organization classification.

**Table 1:** Patient demographics and baseline characteristics

Characteristics	*n* (%) or median (range) *N* = 14
Age (years)	
Median (range)	77.5 (61–84)
≥75	11 (78.6%)
Sex	
Female	3 (21.4%)
Male	11 (78.6%)
ECOG PS	
0	7 (50.0%)
1	6 (42.9%)
≥2	1 (7.1%)
AML type	
*De novo* AML	6 (42.9%)
Secondary AML	8 (57.1%)
Type of secondary AML (*n* = 8)	
Treatment-related	1/8 (12.5%)
Post-MDS	7/8 (87.5%)
AML with MDS-related changes	
Yes	6 (42.9%)
No	8 (57.1%)
Prior systemic therapy	
Prior AZA treatment	5 (35.7%)
Bone marrow blast count	
<30%	8 (61.5%)
≥30% to <50%	4 (30.8%)
≥50%	1 (7.7%)
Missing	1
Baseline neutrophil count (×10^9^/l)	
Median (range)	0.6 (0.0–7.4)
Baseline hemoglobin value (g/l)	
Median (range)	79.5 (66.0–109.0)
Baseline platelet count (×10^9^/l)	
Median (range)	42.0 (14.0–338.0)
RBC transfusion dependence at baseline^a^	
Yes	9 (64.3%)
Platelet transfusion dependence at baseline^a^	
Yes	9 (64.3%)
RBC or platelet transfusion dependence at baseline^a^	
Yes	9 (64.3%)

ECOG, Eastern Cooperative Oncology Group; PS, performance status; AML, acute myeloid leukemia; MDS, myelodysplastic syndrome; AZA, azacitidine; RBC, red blood cell.

^a^Transfusion dependence at baseline was defined as transfusion within 56 days prior to the first dose of study drug.

The median treatment period was 2.0 months (range = 0.7–5.1). All patients discontinued the study treatment. Seven patients (50.0%) continued to receive the same combination treatment using commercially available venetoclax instead of the study drug after its approval. In the other seven patients, the primary reasons for discontinuation included the physician’s decision in three cases (21.4%), PD in two cases (14.2%) and AEs related to and not related to aggravation of AML in one case each (7.1%).

### Safety

TEAEs reported in ≥20% of patients, regardless of severity or relationship to the study drug, are listed in Table 2. All patients experienced at least one grade ≥ 3 TEAE. The most common TEAE (grade ≥ 3) was neutropenia in eight patients (57.1%) followed by other hematological TEAEs, including anemia, lymphopenia, thrombocytopenia and decreased white blood cell count in five patients each (35.7% each), involving patients who experienced multiple TEAEs. Additionally, febrile neutropenia was reported in four patients (28.6%). Any grade of infection was reported in five patients (35.7%), among which two experienced grade ≥ 3 infections, including nasopharyngitis, pneumonia and sepsis (*n* = 1 each) involving a patient who experienced multiple TEAEs.

**Table 2:** Common TEAEs

AEs, ≥20% of patients (VEN + LDAC)	Any grade	Grade 3 or 4
Any TEAEs	14 (100%)	14 (100%)
Hematological AEs		
Neutropenia	8 (57.1%)	8 (57.1%)
Anemia	5 (35.7%)	5 (35.7%)
Decreased WBC count	5 (35.7%)	5 (35.7%)
Lymphopenia	5 (35.7%)	5 (35.7%)
Thrombocytopenia	5 (35.7%)	5 (35.7%)
Leukopenia	4 (28.6%)	4 (28.6%)
Febrile neutropenia	4 (28.6%)	4 (28.6%)
Non-hematological AEs		
Decreased appetite	6 (42.9%)	1 (7.1%)
Nausea	6 (42.9%)	0
Constipation	4 (28.6%)	0
Hypokalemia	3 (21.4%)	1 (7.1%)

AE, adverse event; VEN, venetoclax; LDAC, low-dose cytarabine; TEAE, treatment-emergent AE; WBC, white blood cell.

SAEs were reported in six patients (42.9%) (Table 3). Febrile neutropenia was the most frequently reported SAE (*n* = 3, 21.4%), which was followed by cardiac failure, gastroenteritis, nasopharyngitis, AML (aggravation of the disease) and acute kidney injury (AKI) reported in one patient each. TLS was not reported in this study. The incidence of dose interruption, dose reduction and permanent venetoclax discontinuation owing to TEAEs was 35.7% (*n* = 5), 7.1% (*n* = 1) and 14.3% (*n* = 2), respectively (Table 4). AKI and AML were reported as TEAEs that led to the discontinuation of venetoclax, and heart failure and AML were reported as TEAEs that led to death.

**Table 3:** SAEs of venetoclax and LDAC

SAEs (SOC/PT)	Patients, *N* = 14, *n* (%)
Any SAEs	6 (42.9%)
Blood and lymphatic system disorders	3 (21.4%)
Febrile neutropenia	3 (21.4%)
Cardiac disorder/cardiac failure	1 (7.1%)
Infections and infestations	2 (14.3%)
Gastroenteritis	1 (7.1%)
Nasopharyngitis	1 (7.1%)
Neoplasms: benign, malignant and unspecified/AML^a^	1 (7.1%)
Renal and urinary disorders/AKI	1 (7.1%)

No patients reported tumor lysis syndrome (TLS) in this study, where all patients received either TLS-prophylactic agents or hydration. SAE, serious adverse event; SOC/PT, MedDRA system organ class and preferred term; AKI, acute kidney injury.

^a^MedDRA PT for ‘aggravation of AML,’ which was defined as an investigator-reported AE.

**Table 4:** TEAEs of venetoclax leading to death and action taken

AE (PT) leading to action taken	Patients (*n*) *N* = 14 (100%)
Venetoclax dose interruption, *n* (%)^a^	
Any AEs	5 (35.7%)
Febrile neutropenia	1 (7.1%)
Leukopenia	1 (7.1%)
Neutropenia	1 (7.1%)
Thrombocytopenia	1 (7.1%)
Vomiting	1 (7.1%)
Nasopharyngitis	1 (7.1%)
Decreased WBC count	1 (7.1%)
AKI	1 (7.1%)
Venetoclax dose reduction, *n* (%)^a^	
Any AEs	1 (7.1%)
Leukopenia	1 (7.1%)
Neutropenia	1 (7.1%)
Venetoclax discontinuation, *n* (%)	
Any AEs	2 (14.3%)
AML^a^	1 (7.1%)
AKI	1 (7.1%)
AE leading to death, *n* (%)	
Any AEs	2 (14.3%)
Cardiac failure^b^	1 (7.1%)
AML^b^^,^^c^	1 (7.1%)

AE, adverse event; AML, acute myeloid leukemia; PT, MedDRA preferred term; TEAE, treatment-emergent AE; WBC, white blood cell.

^a^Including patients with multiple AEs.

^b^Cause of death in both cases was considered not to be venetoclax or LDAC in the opinion of the investigator.

^c^MedDRA PT for ‘aggravation of AML,’ which was defined as an investigator-reported AE.

A Grade 3 AKI was observed in an 80-year-old man with hypertension and diabetic nephropathy. He also used nifedipine and furosemide for coronary spastic angina and cardiac failure during study treatment. On Day 24, the antifungal prophylaxis was changed from caspofungin to fluconazole, and the dose of venetoclax was reduced to 300 mg accordingly. Subsequently, elevation of serum creatinine, hypercalcemia and decreased blood pressure were observed. Venetoclax was discontinued on Day 26 owing to persistently elevated creatinine level. Nifedipine and furosemide were also discontinued. On Day 45, renal failure improved. Drug-induced renal injury, decreased blood pressure and hypercalcemia were suspected to be the causes of acute renal failure. The causality of the study drugs could not be ruled out.

Fatal Grade 5 cardiac failure was reported in a 61-year-old male patient during post-treatment. He was previously diagnosed with MDS 75 days before initial dosing of the study drug and was not reported to have received AZA. After his diagnosis of AML, he consented to participate in this study and the Study Cycle 1 was started. On Day 24, bone marrow aspiration showed no therapeutic effect. The study drug was discontinued at the end of Cycle 1 on Day 28. Post-treatment was started on Day 31 with reduced-dose 7 + 3 AraC (67 mg/m^2^ QD for 7 days) and idarubicin (3.4 mg/m^2^ QD for 3 days). The following day, he had pyrexia and dyspnea with decreased oxygen saturation, which led to suspicion of heart failure. On Day 50, oxygen therapy was started due to low oxygen saturation between 89 and 92%. On Day 53, his blood pressure and oxygen saturation levels decreased, and despite intervention, he died. The final administrations of venetoclax and LDAC were >3 weeks and >6 weeks before the onset of the event, respectively. Therefore, this event was considered likely to be associated with the complications of AML and not related to the study treatment.

### Prophylaxis

**Figure 2:**
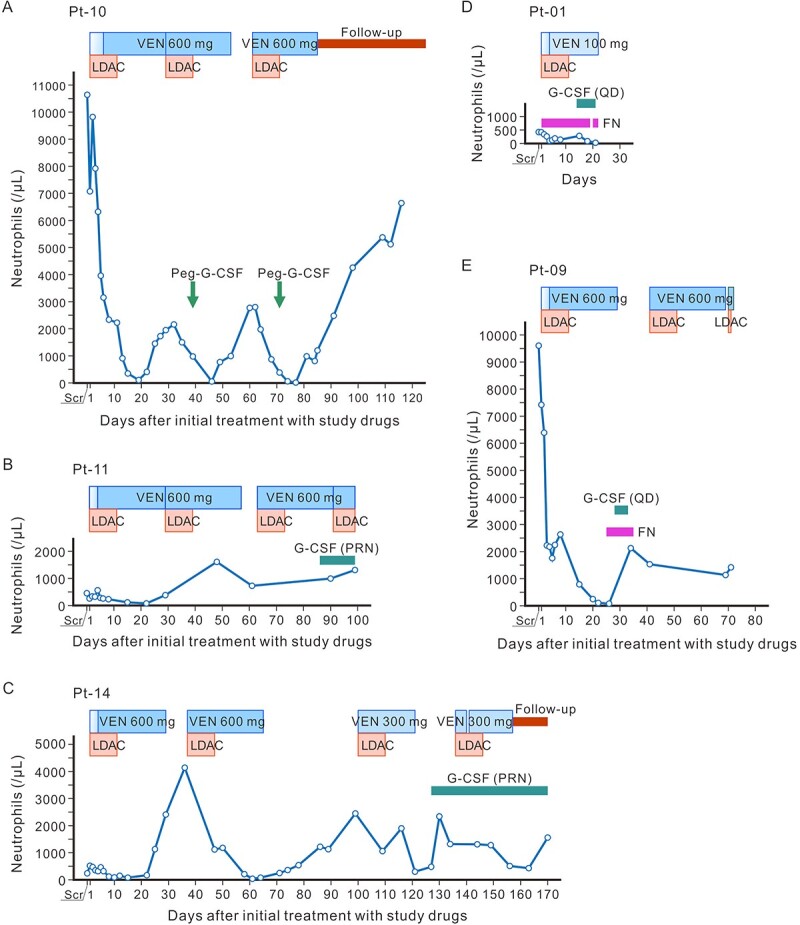
Concomitant use of G-CSF and neutrophil counts. (A–C) Time courses of neutrophil counts in Pt-10, -11 and -14 who received prophylactic G-CSF. (D, E) Pt-01 and -09 received G-CSF as treatment for febrile neutropenia. G-CSF, granulocyte-colony stimulating factor; Peg, pegfilgrastim; FN, febrile neutropenia; PRN, pro re nata; QD, quaque die (once a day or daily); VEN, venetoclax; Pt, patient; Scr, screening.

Anti-fungal agents were concomitantly used in eight patients, mostly as a prophylactic for patients with an absolute neutrophil count <500/μl; namely, fluconazole was used in four patients and voriconazole, micafungin, caspofungin was used for one patient each as antifungal prophylaxis. Dose modification for venetoclax was defined in the protocol when moderate and strong CYP3A inhibitors such as azoles were concomitantly administered. Granulocyte-colony stimulating factor (G-CSF) was used in five patients during venetoclax treatment, and two of them used it during febrile neutropenia (Fig. 2).

### Disease assessment

**Figure 3:**
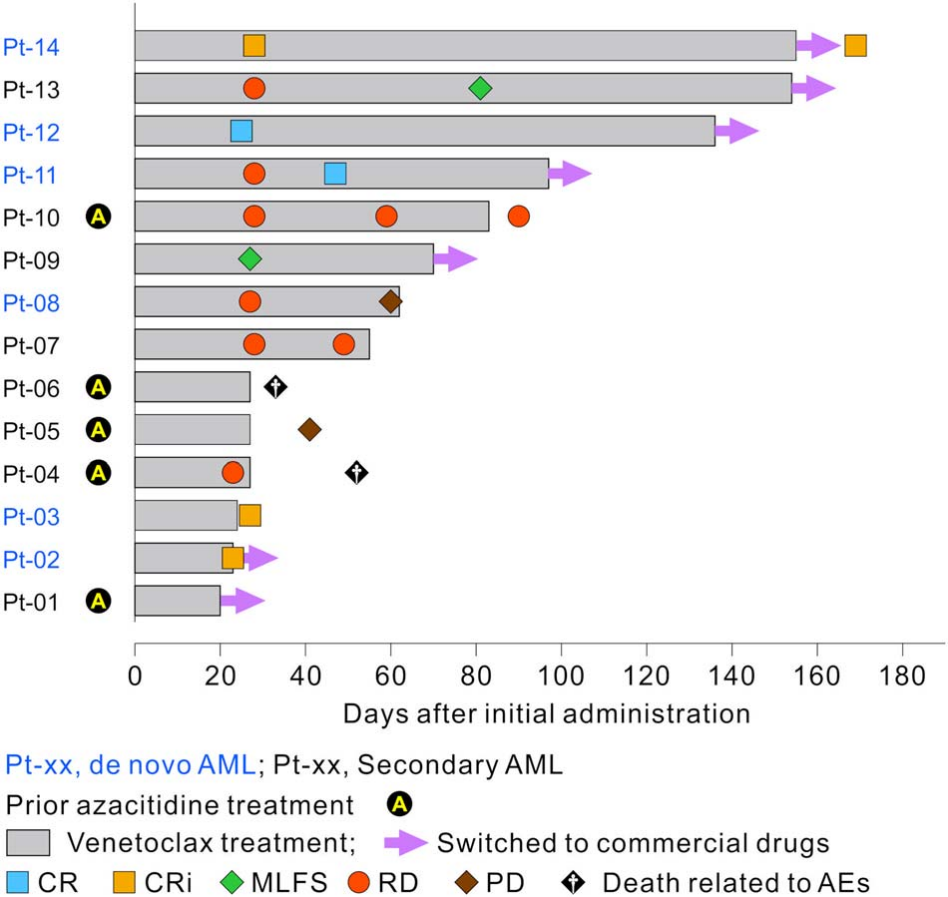
Disease response and treatment duration in each patient, with baseline characteristics. Commercial drugs: patients received commercially available venetoclax + LDAC after approval of venetoclax in Japan. venetoclax treatment, open treatment period; CR, complete remission; CRi, CR with incomplete blood count recovery; MLFS, morphologic leukemia-free state; RD, resistant disease; PD, progressive disease.

**Figure 4:**
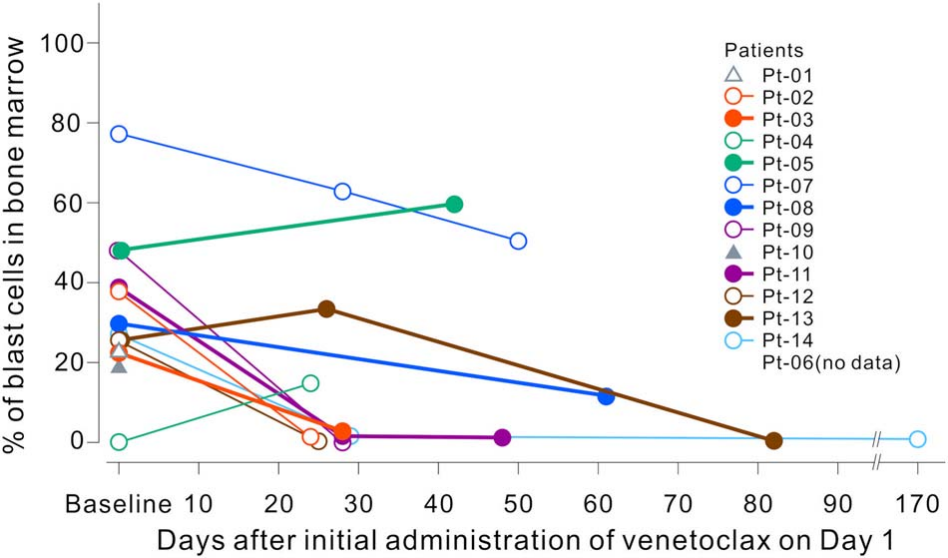
Bone marrow blast count change after the initial administration of venetoclax. Bone marrow aspiration or biopsy for disease assessment was conducted at screening and was done at the investigator’s discretion according to patients’ physical findings or peripheral blood results.

Figure 3 shows responses at assessment time points in each patient. Best responses were CR (*n* = 2), CRi (*n* = 3), morphologic leukemia-free state (*n* = 2), resistant disease (*n* = 4), PD (*n* = 1) and not evaluable (*n* = 1) by investigator assessment. Marrow blasts were decreased from baseline in 10 out of 14 patients, and 7 patients achieved <5% during the study period (Fig. 4). Transfusion independence for red blood cells and platelets was 28.6 and 57.1%, respectively. Total transfusion independence was 28.6%.

## Discussion

In this EAS, venetoclax in combination with LDAC showed a similar safety profile to that in the VIALE-C study. Regarding patient characteristics, the venetoclax plus LDAC arm of the VIALE-C study (*N* = 143) included 82 patients ≥75 years old (57%), 58 patients (41%) with secondary AML, 52 patients (36%) with prior hematologic disorder and 28 patients (20%) who had received prior treatment with hypomethylating agents (AZA or decitabine) for MDS (10). In comparison, the EAS (*N* = 14) included 11 patients ≥aged 75 years (78.6%), 8 patients (57.1%) with secondary AML, 7 patients (50.0%) with an MDS history and 5 (35.7%) who had received prior AZA treatment. These proportions were higher than those in the VIALE-C study, suggesting that patients in the EAS had more severe background disease. The incidence of neutropenia was higher in the patients of this study than in the Japanese subgroup of the VIALE-C study (57.1 vs. 16.7%), but the incidence of febrile neutropenia was lower (28.6 vs. 50.0%) (5). TLS was not reported in this EAS; it is thought to be manageable using risk mitigation measures, including appropriate prophylaxis and monitoring.

The patient with AKI developed renal failure and decreased blood pressure after changing the antifungal prophylaxis to fluconazole. Fluconazole, which is a moderate cytochrome P450 3A (CYP3A) inhibitor, may have increased the blood concentration of nifedipine, which is a substrate of CYP3A, and decreases blood pressure and could have resulted in prerenal failure. Additionally, the patient’s history of diabetic nephropathy may have contributed to the development of AKI. In the VIALE-C study, 7 out of 142 patients (4.9%) in the venetoclax + LDAC group had AKI (including 1 with SAE) compared with 5 of 68 patients (7.4%) in the placebo + LDAC group, with no increase in venetoclax treatment, although no SAEs were reported in the latter group (16). Venetoclax is mainly metabolized by CYP3A in the liver, and <0.1% is excreted into the urine (17). Therefore, the risk of renal injury is presumed to be low. However, coadministration of venetoclax with CYP3A and/or P-glycoprotein inhibitors increases venetoclax blood concentrations and requires a dose reduction of venetoclax (18,19). Because older patients often have co-morbidities and use multiple medications, drug interactions should be carefully monitored. In addition, it should also be noted that Ca antagonists, which are frequently used as antihypertensive drugs in Japan, are also metabolized by CYP3A; thus, caution is needed when Ca antagonists are coadministered with CYP3A inhibitors, which is similar to venetoclax (20,21).

G-CSF was used in 5 out of 14 patients (35.7%), including for prophylactic purposes (*n* = 3). Among these, one patient received pegylated G-CSFs after LDAC administration (22). The use of G-CSF may have contributed to the lower incidence of febrile neutropenia (28.6% in this EAS vs. 50.0% in the Japanese VIALE-C population) despite the higher incidence of neutropenia (57.1 vs. 16.7%), although this cannot be confirmed owing to the coincided drug holiday and the small sample size in both studies. Furthermore, neither study was designed to assess the effect of G-CSF, and its use was not mandated by the protocol.

This study did not include efficacy endpoints because this was an EAS focusing on ‘early access’ for patients with no effective therapy; however, bone marrow assessment after Cycle 1 was performed to evaluate the disease condition at the discretion of each investigator. The rates of CR and CRi appeared to be similar to the outcomes of the VIALE-C study. Additionally, the achievement rate of transfusion independence suggested efficacy of venetoclax plus LDAC, which is comparable with that in the VIALE-C study (11).

The VIALE-C study confirmed the benefit–risk balance of venetoclax treatment in combination with LDAC among patients with untreated AML who are ineligible for intensive chemotherapy and for whom treatment options are limited (10). This study provided supportive data regarding the benefit–risk balance of venetoclax treatment in combination with LDAC, more closely applicable to the real-world clinical treatment of Japanese patients with AML than that in the VIALE-C study. This EAS study included more older patients and patients with secondary AML than the VIALE-C study, highlighting the fact that the patient background will be more serious in actual clinical practice. These results indicate that it is important to pay close attention to complications and concomitant medications during this treatment.

In conclusion, the AEs reported in this EAS were consistent with the known safety profile of venetoclax plus LDAC and were successfully managed with standard medical care. The findings of this EAS further support the benefit–risk profile of venetoclax plus LDAC shown in the VIALE-C study.

## Abbreviations

AE, adverse event; AKI, acute kidney injury; AML, acute myeloid leukemia; AraC, cytarabine; AZA, azacitidine; CR, complete remission; CRi, complete remission with incomplete blood count recovery; CYP3A, cytochrome P450 3A; EAS, expanded access study; G-CSF, granulocyte-colony stimulating factor; LDAC, low-dose cytarabine; MDS, myelodysplastic syndrome; PD, progressive disease; PS, performance status; QD, once a day or daily; SAE, serious adverse event; TEAE, treatment-emergent adverse event; TLS, tumor lysis syndrome

## Data sharing and data accessibility statement

AbbVie is committed to responsible data sharing regarding the clinical trials we sponsor in which this EAS is contained. This includes access to anonymized, individual- and trial-level data (analysis data sets) as well as other information (e.g. protocols and Clinical Study Reports or analysis plans) as long as the trials are not part of an ongoing or planned regulatory submission. This includes requests for clinical trial data for unlicensed products and indications.

These clinical trial data can be requested by any qualified researchers who engage in rigorous, independent scientific research and will be provided following review and approval of a research proposal and statistical analysis plan and execution of a data sharing agreement. Data requests can be submitted at any time after approval in the US and Europe and after acceptance of this manuscript for publication. The data will be accessible for 12 months, with possible extensions considered. For more information on the process, or to submit a request, visit the following link: https://www.abbvie.com/our-science/clinical-trials/clinical-trials-data-and-information-sharing/data-and-information-sharing-with-qualified-researchers.html.
